# Respiration‐Triggered Release of Cinnamaldehyde from a Biomolecular Schiff Base Composite for Preservation of Perishable Food

**DOI:** 10.1002/advs.202306056

**Published:** 2023-12-21

**Authors:** Fei Liu, Lingyun Kuai, Chen Lin, Maoshen Chen, Xing Chen, Fang Zhong, Tao Wang

**Affiliations:** ^1^ School of Food Science and Technology Jiangnan University Wuxi 214122 China; ^2^ National Engineering Research Center for Cereal Fermentation and Food Biomanufacturing Jiangnan University Wuxi 214122 China; ^3^ International Joint Laboratory on Food Safety Jiangnan University Wuxi 214122 China; ^4^ Jiaxing Institute of Future Food Jiaxing 314050 China; ^5^ Science Center for Future Foods Jiangnan University Wuxi 214122 China

**Keywords:** biomolecular preservatives, food preservation, respiration‐responsive, Schiff base, self‐saving

## Abstract

One‐third of the food produced worldwide is wasted annually and never consumed, of which ≈ 40–50% are perishable vegetables and fruits (VFs). Although various methods are proposed to reduce this loss, high manufacturing costs and food safety concerns pose significant challenges for the preservation of VFs. Herein, a respiration‐triggered, self‐saving strategy for the preservation of perishable products based on a biomolecular Schiff base composite fabricated by imidization of chitosan and cinnamaldehyde (CS‐Cin) is reported. Ripening of VFs produces acid moisture and triggers a Schiff base reaction in CS‐Cin, permitting the release of volatile Cin into the storage space. This enables versatile preservation by placing CS‐Cin on the side without the need to touch the food, like the desiccant packet in a food packaging bag, while the rotting of VFs is retarded in a self‐saving manner. As a result, the lifetimes of broccoli and strawberries are extended from 2 to 8 days. Furthermore, CS‐Cin with restored preservative properties can be repeatedly recycled from used CS via imidization with Cin. Compared with conventional techniques, the preservatives are easy to use, versatile, and cost‐effective, and the respiration‐responsive release of Cin empowers a self‐saving approach toward the smart preservation of perishable food.

## Graphical abstract

A biomolecular Schiff‐base composite is fabricated by imidizing chitosan and cinnamaldehyde (CS‐Cin). Ripening fresh produce releases acidic moisture and triggers the Schiff base reaction in CS‐Cin, permitting the release of volatile Cin into the storage space. This enables a versatile non‐contact preservation strategy in a self‐saving and recyclable manner with high cost‐effectiveness.

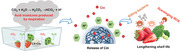

## 1 Introduction

More than 10% of the world's population is experiencing famine and undernourishment.^[^ ^1^ ^]^ However, an astonishing fact is that one‐third of the food produced worldwide, that is, ≈15 million tons annually, is wasted and never consumed, of which ≈40−50% are perishable vegetables and fruits (VFs) that are lost in vain.^[^ ^1^, ^2^ ^]^ With the unremitting war and the prognosis of the COVID‐19 pandemic, the extension of food transportation and distribution from farms to retailers has further deteriorated food spoilage. Furthermore, 15 billion m^3^ water footprint and 20 megatons of CO_2_eq greenhouse gas emissions are associated with food waste annually.^[^ ^3^ ^]^ Therefore, it is imperative to develop advanced strategies for versatile and effective lengthening of the lifetime of perishable food.

The rotting of fresh food is closely associated with microbial growth and respiration‐related peroxidative damage, ultimately leading to dehydration and senescence.^[^ ^4^ ^]^ Various methods have been developed to inhibit pathogenic invasion and respiration in fresh VFs. For example, fruit waxing is a commercial technique that forms conformal coatings on fresh produce^[^ ^5^ ^]^ where the inorganic acid coating forms a physical barrier preventing microbial growth and resisting fruit respiration.^[^ ^1a^ ^]^ This has inspired the development of many other coating‐based materials, such as antibacterial coatings carrying food‐grade preservatives.^[^ ^6^ ^]^ Another coating approach is to regulate gas (O_2_, CO_2_, and H_2_O) permeability and CO_2_/O_2_ selectivity using a membrane that mimics the stomata of plant leaves.^[^ ^7^ ^]^ Despite such progress, the coating strategy is highly exclusive to VFs with relatively smooth surfaces with low local curvatures, such as oranges and mangoes. However, for those with rough, gullied surfaces such as broccoli with crowded buds, the high capillary forces of the coatings created at the rim of neighboring buds prevent the formation of coherent films with sufficient conformity. Non‐contact preservation methods such as refrigeration, irradiation, and modified atmospheric packaging are also prevalent and have been applied in the preservation of such fresh produce with a surface resistant to preservative coating as broccoli and strawberries.^[^ ^8^ ^]^ However, these methods are expensive and require the use of specific facilities, which not only cannot be applied to the preservation of cheap agricultural products, but also complicates the food supply chain and reduces cost‐effectiveness.

**Figure 1:**
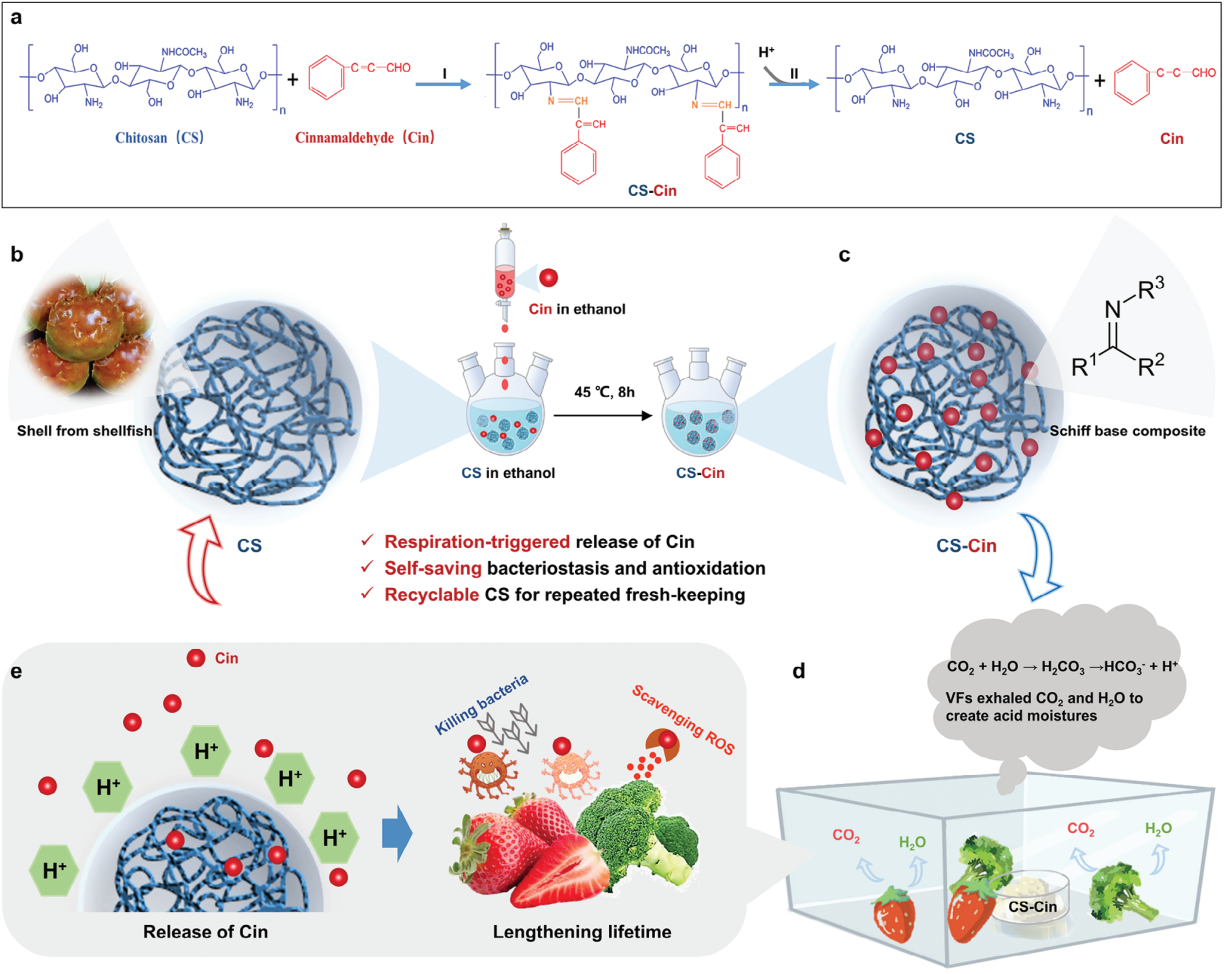
Chemical basis and experimental design of the respiration‐triggered self‐saving strategy for preservation of perishable food. a) Assembly of CS‐Cin via imidization between amino groups of CS and aldehyde groups of Cin (reaction I) and disassembly of CS‐Cin via Schiff base interaction (reaction II). b,c) The CS was extracted from discarded shell of shellfish, which was used as the substrate of Cin for the assembly of CS‐Cin using a benign condition at 45 °C for 8 h. d) Schematic illustration of preservation of perishable VFs using CS‐Cin as a side without touching the perishable food. The respiration of VFs exhaled CO_2_ and H_2_O to create acid moisture. e) The acid moistures triggered the Schiff base reaction in CS‐Cin to release Cin that in turn suppressed the microbial growth and peroxidation, leading to weakened respiration and lengthened lifetime of VFs. The CS can be reused after releasing Cin using the same procedure as the assembly of fresh CS‐Cin, contributing to recyclable preservatives for repeated fresh‐keeping.

Herein, we report a versatile, respiration‐triggered, self‐saving strategy for the preservation of perishable products based on a biomolecular Schiff base composite fabricated by the imidization of chitosan and cinnamaldehyde (CS‐Cin) (**Figure** **1**a, reaction I). CS is an amino‐bearing biopolymer found in shellfish. The rich amino groups in CS enable its facile imidization with aldehydes toward the formation of the Schiff base, where the imine groups are susceptible to acid ions, triggering the release of aldehydes.^[^ ^9^ ^]^ Cin is a volatile essential oil extracted from the bark of cinnamon trees. The inherent antibacterial and antioxidative properties of Cin make it a promising candidate for preserving VFs and meat.^[^ ^10^ ^]^ CS‐Cin was synthesized under benign conditions by dropwise addition of a Cin solution (dissolved in ethanol) to a CS dispersion (dispersed in ethanol) at 45 °C for 8 h (Figure 1b).^[^ ^9b^ ^]^ The assembly was reversible, and disassembly (Schiff base reaction) occurred when acid ions were brought to the Schiff base, leading to the reformation of CS and Cin (Figure 1a, reaction II). This enabled preservation by placing the preservatives on the side without the need to touch the food (Figure 1d), similar to the desiccant packet in a food packaging bag, yet a smart, spontaneous preservation process was initiated by acid moisture resulting from food respiration that triggered the Schiff base reaction, permitting the release of Cin to the storage space (Figure 1e). As a result, bacterial invasion and peroxidative damage were retarded by Cin, leading to weakened respiration, delayed rotting, and lengthened lifetime (up to 300%) of VFs. Furthermore, after the release of Cin, the used CS repeatedly reacted with Cin to regenerate CS‐Cin (Figure 1b) with restored preservative properties. Although CS and Cin have been used for the development of food‐preserving materials such as films, emulsions, and nanoparticles,^[^ ^11^ ^]^ coating or wrapping of food is required during food preservation. This is the first study reporting the non‐contact preservation of perishable food by CS‐Cin featuring rational management of Cin release as feedback of food respiration in a self‐saving manner, which shows considerable advantages over existing methods in terms of cost‐effectiveness ($0.02–0.03 kg^−1^ food), convenience, recyclability, and versatility.

## 2 Results and Discussion

### 2.1 Formation of CS‐Cin Bearing Schiff Base Moieties

**Figure 2:**
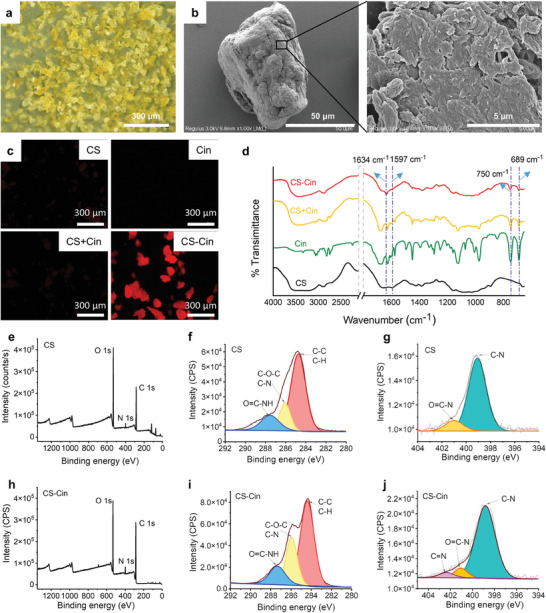
Characterization of CS‐Cin. a) Optical microscopy of CS‐Cin powders. b) SEM images of a representative CS‐Cin particle. c) Fluorescence image of samples, where red fluorescence indicates the existence of Schiff base composites. d) FT‐IR spectra. e) XPS spectra of CS. f,g) High‐resolution f) C1s, and g) N1s spectra of CS. h) XPS spectra of CS‐Cin. i,j) High‐resolution i) C1s and j) N1s spectra of CS‐Cin.

CS presented as yellow particles with an average diameter of 50 µm (**Figure** **2**a). Scanning electron microscope (SEM) revealed that the particles had irregular geometries, featuring rough architectural topologies with high surface areas (Figure 2b). The formation of CS‐Cin bearing Schiff base moieties was intuitively visualized by excitation at 488 nm using a fluorescence microscope.^[^ ^12^ ^]^ Figure 2c shows that neither CS nor Cin exhibited visible emission when scanned in the red channel (570–600 nm). In contrast, CS‐Cin exhibited strong red fluorescence associated with the formation of imine groups. We also performed a controlled study by dropping liquid Cin onto the surface of CS at room temperature. Fluorescence imaging revealed that the control (CS + Cin) did not emit red fluorescence, indicating that imidization did not occur under these mild conditions.

Figure 2d shows that CS had characteristic Fourier transform infrared (FT‐IR) bands at 1597, 1655, and 3200–3600 cm^−1^, attributed to the bending vibrations of amino groups (‐NH_2_/‐NH), stretching vibrations of amide I groups (‐C═O), and overlapping signals of the stretching vibrations of ‐OH and ‐NH groups, respectively.^[^ ^13^ ^]^ The characteristic bands of Cin were located at 1681, 1625, and 1495 & 1449 cm^−1^, corresponding to the stretching vibrations of the C═O groups, stretching vibrations of the C═C groups, and stretching vibrations of the benzene rings, respectively.^[^ ^11a^ ^]^ No new visible FT‐IR bands were observed for CS + Cin, indicating negligible reactions between CS and Cin, which agreed well with the results of fluorescence microscopy (Figure 2c). In contrast, apart from the abovementioned characteristic bands appearing in CS and Cin, a new band at 1634 cm^−1^ emerged in CS‐Cin, corresponding to the stretching vibrations of the imine groups (C═N).^[^ ^14^ ^]^ Because the formation of the Schiff base consumes amino groups, the band of their bending vibrations at 1597 cm^−1^ disappeared, whereas the stretching vibrations of O─H and the vibrations of glucosidic bonds alongside the main chains remained unchanged. These results indicated that imidization occurred at the amino sites of the CS side chains. To determine whether ‐NH_2_ or ‐NH (acetamido–NHCOCH_3_) dominated the reactions, we used CS with different levels of deamidization (80%−90%) as the substrate of Cin. As the deamidization of CS corresponds to the formation of ‐NH_2_ at the expense of ‐NH, CS with higher levels of deamidization resulted in stronger band intensities of the imine groups (Figure S1, Supporting Information), highlighting the realization of imidization between the ‐NH_2_ groups of CS and the aldehyde groups of Cin.

The imidization was further studied using X‐ray photoelectron spectroscopy (XPS) (Figure 2e,h). After peak‐fitting of C1s (286 eV), the high‐resolution spectra of CS and CS‐Cin showed three peaks at 284.68, 286.08, and 287.68 eV (Figure 2f,i), corresponding to C‐C/C‐H, C‐O‐C/C‐N, and CONH, respectively.^[^ ^15^ ^]^ Because of the bonding of Cin and CS, the CS‐Cin had stronger binding energy at 286.08 eV than that of individual CS. The quantitative results verified that the relative amount of C‐O‐C/C‐N for CS‐Cin (34.66%) was higher than that for CS (20.5%) (Table S1, Supporting Information). The peak‐fittings of N1s (399 eV) suggested that the N atoms existed in the forms of C‐NH_2_ and ‐NHCOCH_3_ in CS (Figure 2g), corresponding to binding energies at 399.08 and 401.08 eV, respectively.^[^ ^16^ ^]^ However, a new electron peak at 402.38 eV appeared for CS‐Cin (Figure 2j), confirming the formation of imine groups,^[^ ^17^ ^]^ which agreed with the FT‐IR results (Figure 2d). Nevertheless, the electron intensity of ‐NHCOCH_3_ in CS was similar to that in CS‐Cin, indicating that imidization occurred at the amino groups instead of the acetamido groups of CS.

### 2.2 Respiration‐Triggered Preservation of Food

**Figure 3:**
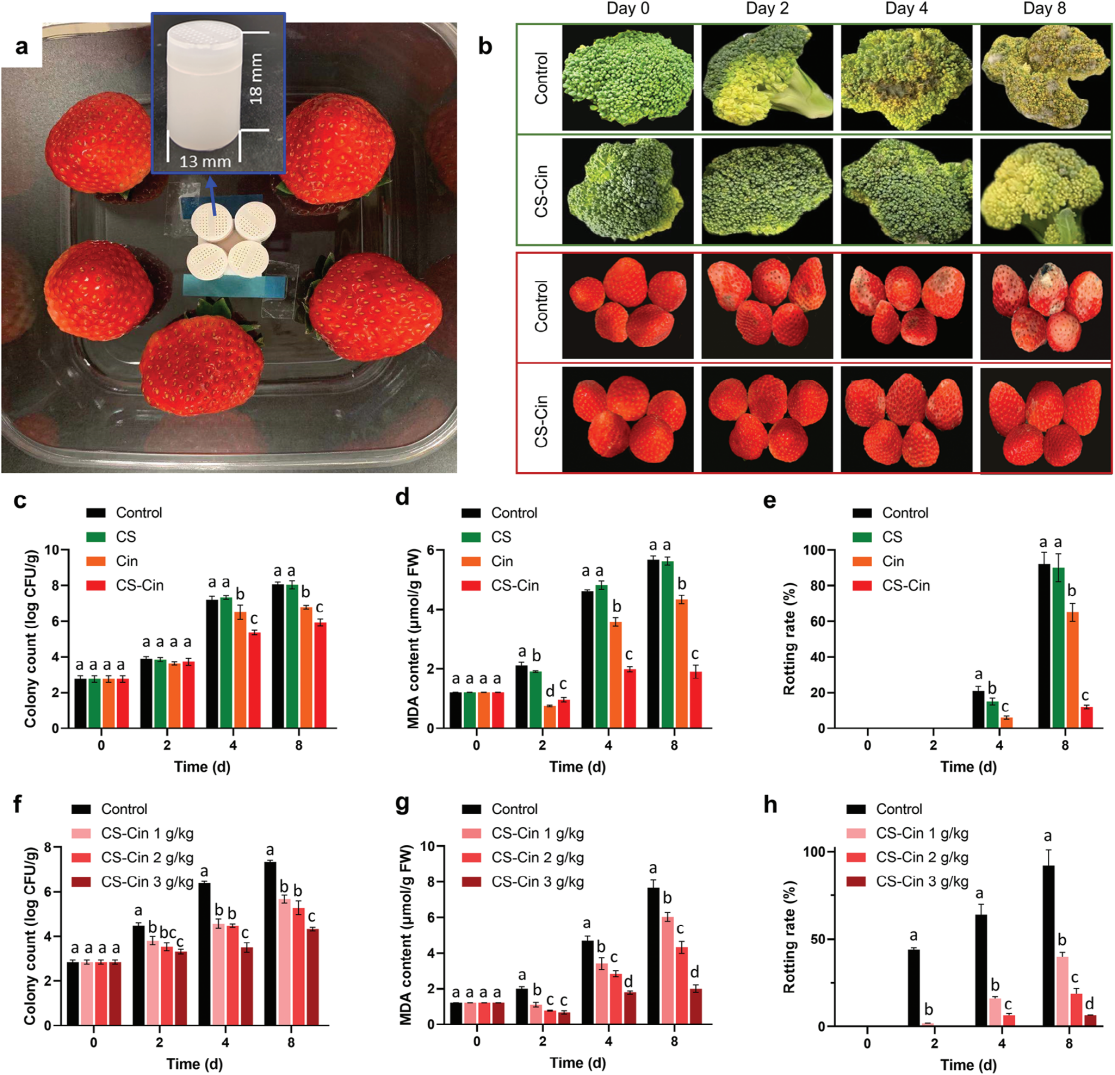
Lengthening of lifetime of VFs by CS‐Cin. a) Top‐view of the preservation box in which the preservative packet was fixed at the center and VFs were placed around. b) Optical images of VFs stored for different durations with or without (control) CS‐Cin. c–e) Time‐dependent c) Colony count, d) MDA content, and e) Rotting rate of broccoli with a CS‐Cin usage of 2 g kg^−1^ VFs. f–h) Time‐dependent f) Colony count, g) MDA content, and h) Rotting rate of strawberries with varied CS‐Cin usages. The CS and Cin groups had equivalent amounts of CS and Cin to the CS‐Cin group, respectively. Different letters among counterparts indicate significant differences (*p* < 0.05).

Both broccoli and strawberries are perishable VFs with a high respiration level, quickly decaying within 2–3 days. Moreover, the preservation of both fresh produce types is challenged by irregular topologies of the surfaces, that is, broccoli has crowded protruded buds, whereas strawberries have numerous cupped pits. Applying a coating strategy is challenging because both the bud and pit structures feature high curvatures that create high capillary forces, preventing the uniform spread of coatable preservatives. In one specific experiment, the CS‐Cin particles were placed in a ventilated packet fixed at the center of a polyvinyl chloride (PVC) plastic container (Figure S2, Supporting Information), and the VFs, e.g. strawberry fruits, were located around the preservatives at 25 °C (**Figure** **3**a). The protocol is scalable because larger containers with more CS‐Cin can be used to preserve bulky food such as broccoli.

The preservation experiments were divided into four groups as follows: a control group with an empty preservative packet, a CS group with CS in the preservative packet, a Cin group with Cin in the preservative packet, and a CS‐Cin group with CS‐Cin in the preservative packet. During long‐term storage, the broccoli control group demonstrated a rapid yellowing process from the 2nd day after purchase, while obvious mildew growth and rotting occurred on the 4th day after purchase. Complete rotting (>90%) occurred after an 8‐day storage after purchase. In contrast, in the CS‐Cin group, yellow broccoli appeared on the 8th day after purchase, with invisible rottenness. Whitening is a typical decay phenomenon in strawberries, which occurred on the 2nd day after purchase in the control group, and substantial whitening and apparent mildew growth were observed on the 8th day after purchase. In contrast, only slight whitening spots were observed in the CS‐Cin group after an 8‐day storage. The results highlighted that a 6‐day or 300% extension of the lifetime was realized for both broccoli and strawberries. Although CS is antibacterial, the CS group presented a parallel rotting process to that of the control group because a non‐contact preservation strategy was used in this study (Figure 3b; Figure S3, Supporting Information). The Cin group also failed to effectively lengthen the lifetime of broccoli owing to rapid volatilization or consumption by the VFs (Figure S3, Supporting Information).^[^ ^18^ ^]^

The colony count of VFs reflects their immune ability against bacterial invasion. The control and CS groups had similar time‐dependent colony counts during storage, which increased by 191% on the 8th day after purchase compared to their newly purchased counterparts (Figure 3c). Cin reduced the 8‐day growth of the colony count by 46% during preservation. Cin droplets quickly evaporated in 1 day, which could not impart sustained bacteriostasis during the 8‐day storage. However, only 50−60% of Cin was released from CS‐Cin after triggered release for 5 days under acidic conditions (Figure 5d). Therefore, microbial growth was further reduced by 31% in the CS‐Cin group, verifying that the respiration of fresh food triggered the rational release of Cin for the prolonged prevention of bacterial growth.

The levels of malondialdehyde (MDA) in VFs symbolize the peroxidation levels of lipid membranes and injury levels of the membrane systems. Higher levels of MDA indicate higher levels of cytomembrane injury, which accelerates apoptosis.^[^ ^19^ ^]^ Moreover, the accumulation of MDA leads to the malfunction of organelles. Figure 3d shows that the control and CS groups demonstrated rapid accumulation of MDA during storages of VFs. Cin reduced MDA growth to some extent owing to its antioxidative abilities, whereas CS‐Cin markedly reduced 8‐day MDA levels by 66%. Furthermore, the respiration rate and weight loss showed the same time‐dependent variations as the MDA data in the control, CS, Cin, and CS‐Cin groups (Figure S4, Supporting Information). Because peroxidation and respiration are closely related to the senescence of VFs, the sharply reduced MDA levels and respiration rates resulted in a greatly lowered rotting rate of the VFs. In sharp contrast to the control groups, where over 90% of the VFs were rotted after 8‐day storage, the rotting rates were reduced to 12% and 6% for broccoli and strawberries stored with 2 g CS‐Cin per kg VFs, respectively (Figure 3e,h).

Moreover, the fresh‐keeping abilities can be tailored by varying the amount of CS‐Cin in the preservative packet. Figure 3f–h and Figure S5 (Supporting Information) show that the colony count, MDA, rotting rate, respiration rate, and weight loss of strawberries were reduced with increasing CS‐Cin, and up to 94% of strawberries survived the 8‐day storage. The results showed that a 6‐day extension (from 2 to 8 days) of the lifetime was achieved for both broccoli and strawberries. As the lifetime of both VFs was less than 2 days, such a 300% extension in lifetime would endow a profound lengthening of their freshness during transportation and marketing.

### 2.3 Regeneration and Repeated Uses of CS

**Figure 4:**
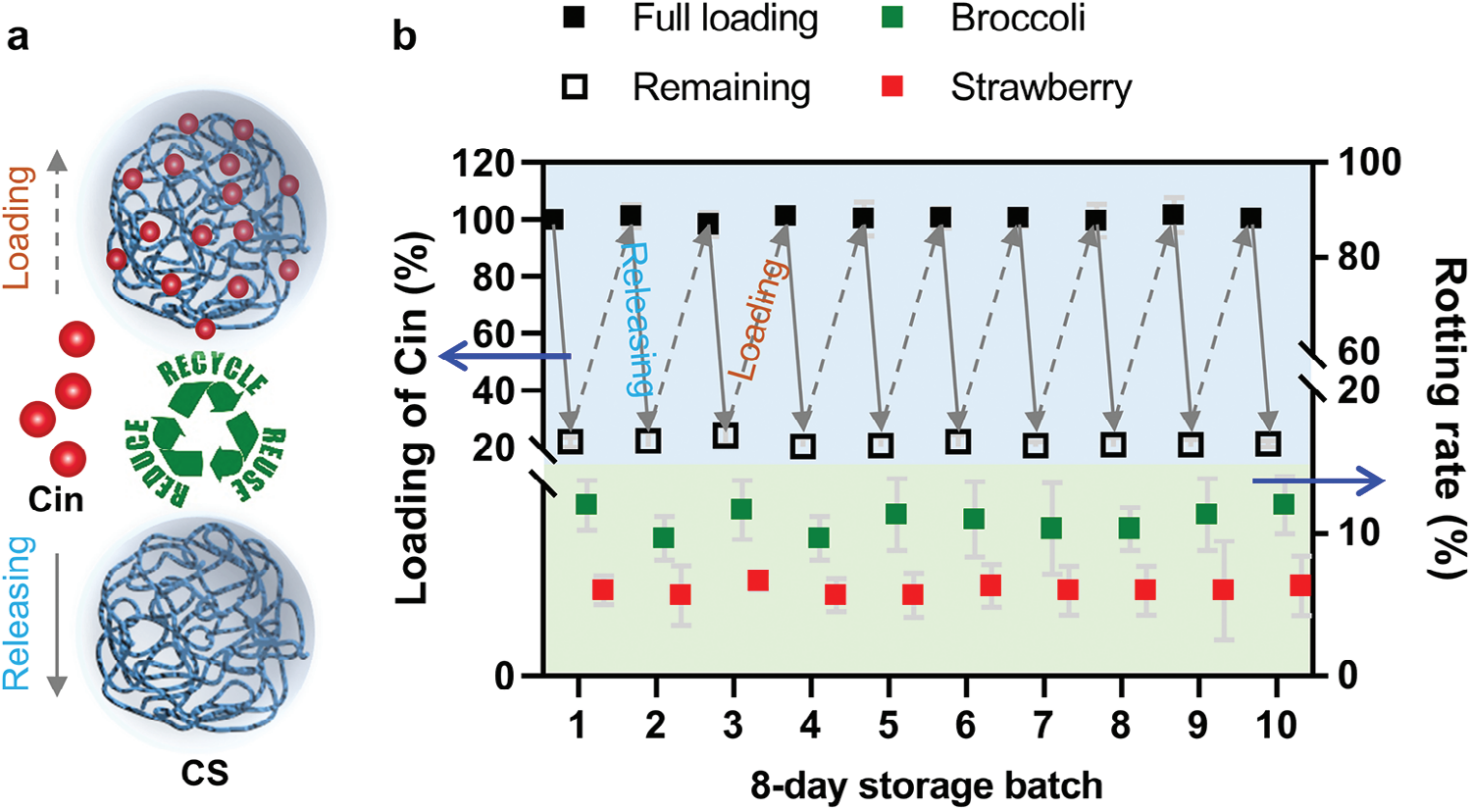
Regeneration of CS for repeated 8‐day preservation of VFs. a) Schematics of loading and releasing of Cin by CS. b) Amounts of Cin in CS and rotting rate of VFs during 8‐day preservations with cyclic regeneration of CS‐Cin. The loading data were normalized to Cin% in the freshly prepared CS‐Cin.

The Cin content in CS‐Cin was lowered by 78%, as determined by measuring the remaining Cin in CS‐Cin after preserving one batch of VFs for 8 days. The CS was then collected and subjected to imidization with fresh Cin to reassemble CS‐Cin for follow‐up preservation (**Figure** **4**a). Such preservation and regeneration could be repeated 10 times with invariable loadings of Cin and similar preservative abilities of CS‐Cin (Figure 4b).

Taken together, this study demonstrates the respiration‐triggered control over preservative release, which in turn modulates the respiration and ripening of VFs, rendering a facile self‐saving strategy for the smart preservation of VFs. The recyclability of CS‐Cin significantly enhanced the cost‐effectiveness, and only an additional $0.02–0.03 kg^−1^ food was imposed for a 300% extension of the lifetime of perishable produce.

### 2.4 Simulation of Respiration‐Triggered Release of Cin

**Figure 5:**
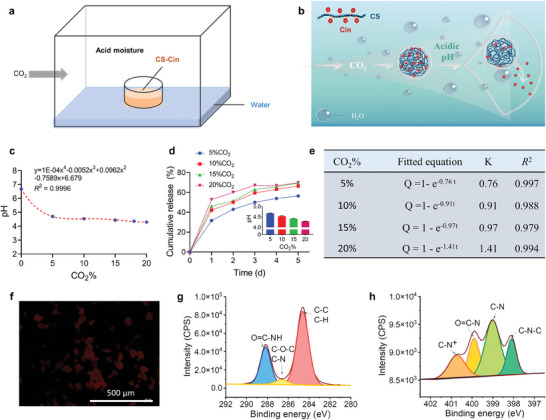
Release of Cin from CS‐Cin triggered by acid moistures. a) Schematics for creating acid moisture by filling CO_2_ of different concentrations in a sealed container in which CS‐Cin is fixed at the center and distilled water is spread over the bottom. b) Schematics of release of Cin from CS‐Cin due to Schiff base reaction triggered by the acid moistures. c) CO_2_ concentration‐dependent pH of the distilled water after sealing with CO_2_ for 5 days. d) Time‐dependent release of Cin. The inset shows the water pH after sealing with CO_2_ for 5 days. e) Parameters of the Cin release profiles fitted with a first‐order release kinetics. f) Fluorescence imaging of CS‐Cin after allowing release of Cin in the acid moistures for 5 days. The faded red fluorescence indicated disassembly of the CS‐Cin. g,h) High‐resolution g) C1s and h) N1s spectra of CS‐Cin after allowing release of Cin in the acid moistures for 5 days.

To mimic the respiration‐triggered Schiff base reaction, the CS‐Cin packet was fixed at the center of a container, in which distilled water was spread over the bottom to create a moisturized atmosphere (**Figure** **5**a). Then, the container was filled with 5–20% CO_2_ which acidified the moisture, triggering the release of Cin from CS‐Cin (Figure 5b). Figure 5c shows that the water pH was reduced in a CO_2_ concentration‐dependent manner after sealing with CO_2_ for five days. Meanwhile, CS‐Cin showed a two‐stage release profile, that is, a fast release within 2 d and a plateau release thereafter (Figure 5d). The release rate in the fast release stage increased with increasing CO_2_ concentration, corresponding to a decreased moisture pH. The percentage of cumulative release (*Q*%) of Cin with time (*t*) complied with first‐order kinetics, that is, *Q*% = 1 − e^−^ *^kt^* (*R* ^2^ > 0.98), where *k* is the release rate constant. As reflected by the *k* values, higher CO_2_ concentrations resulted in faster Cin release rates (Figure 5e). Therefore, the preservation ability of CS‐Cin can be modulated by the respiration of VFs, enabling the development of a smart self‐saving preservation strategy.

After allowing the reaction with the acid moisture for 5 days, the intrinsic fluorescence of the preservatives faded away (Figure 5f), indicating the release of Cin from CS‐Cin. High‐resolution C1s XPS revealed that the binding energy of CS‐Cin (286.08 eV) was lower than that of its freshly prepared counterparts (Figure 5g), indicating a decrease in the relative amounts of C‐O‐C/C‐N. In addition, compared to the freshly prepared CS‐Cin, the high‐resolution N1s spectra of treated CS‐Cin illustrated a shift of the binding energy from 402.48 to 400.78 eV (Figure 5h), characteristic of the formation of C‐N^+^ at the expense of C═N. These results confirmed the acid moisture‐triggered Schiff base reactions in CS‐Cin, resulting in the release of Cin in an acidity‐dependent manner.

### 2.5 pH‐Responsive Antibacterial Properties of CS‐Cin

**Figure 6:**
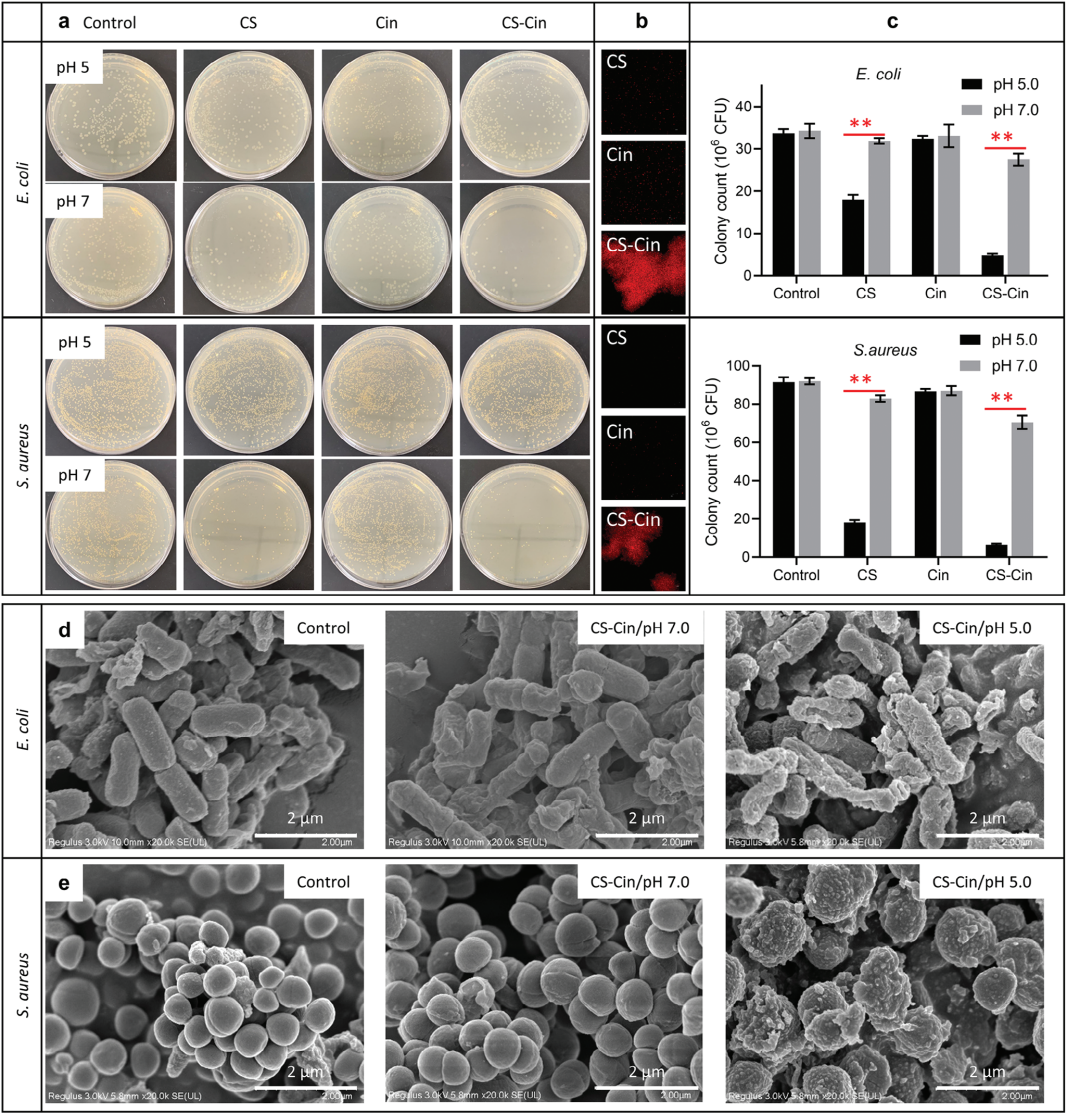
Antibacterial properties of CS‐Cin against *E. coli* and *S. aureus*. a) Images of *E. coli* and *S. aureus* colonies cultured in petri dish. b) Fluorescence images of *E. coli* and *S. aureus* dyed with PI that can ignite red fluorescence when combined with dead bacteria. c) Colony counts *E. coli* and *S. aureus* at pH 5.0 and pH 7.0. ^**^ indicates differences at *p* < 0.01. d,e) SEM images of d) *E. coli* and e) *S. aureus*.

CS and Cin are antibacterial agents widely used for food preservation.^[^ ^6^, ^20^ ^]^ In this study, two bacteria models, i.e. *Escherichia coli (E. coli)* and *Staphylococcus aureus (S. aureus)*, were used to test the pH‐responsive antibacterial properties of CS‐Cin. Each bacterial model was cultured in media (pH 5.0, pH 7.0) supplemented with bare PBS (control), PBS containing CS (1 mg mL^−1^), PBS containing Cin (0.25 mg mL^−1^), and PBS containing CS‐Cin (1.25 mg mL^−1^). After culturing for 24 h, the visual appearances of the colonies were photographed and counted, and the morphologies of the bacteria were examined using SEM. From the results of colony plates, the control group of either *E. coli* or *S. aureus* presented commensurable colony counts between pH 7.0 and pH 5.0, indicating that both bacteria were alive under the tested conditions. The Cin groups had similar live bacterial colonies to those of the control at either pH 7.0 or pH 5.0 (**Figure** **6**a). Although Cin has antibacterial properties, the low water solubility (1.42 mg L^−1^) and low concentration (0.25 mg mL^−1^) used in this study may have prevented effective internalization of Cin into the bacteria.^[^ ^21^ ^]^ The colony counts of the CS group at pH 7.0, which were reduced to an even lower extent at pH 5.0, were lower than those of the control. The antibacterial properties of CS result from its positive charges, which can bind to negatively charged mucosal layers.^[^ ^22^ ^]^ Acidification‐induced protonation of the amino group of CS enabled the transformation of ‐NH_2_ to ‐NH_3_^+^, causing injury to the bacterial mucosal layers. Interestingly, CS‐Cin elicited stronger bacteriostasis against *E. coli* or *S. aureus* than CS or Cin alone at pH 7.0, which may be due to the increased contact of bacteria with Cin, in addition to bacteriostasis by CS. By increasing the acidity to pH 5.0, most of the bacteria were killed (>86% killing rate) because of the triggered release of Cin from CS toward the bacteria that electrostatically combined with CS.

Dead bacteria were stained with propidium iodide (PI), which emits red fluorescence when combined with the apoptotic nuclei.^[^ ^23^ ^]^ Figure 6b shows that the control groups of both *E. coli* and *S. aureus* exhibited negligible fluorescence, indicating good viability of both bacteria. This was also confirmed by the quantitative data (Figure 6c). Although CS‐Cin exerted bacteriostasis against *E. coli* and *S. aureus* at pH 7.0, the 9.21%−13.04% antibacterial rate (Figure 6c) was unable to ignite sufficient PI red fluorescence (Figure 6b). In contrast, CS‐Cin demonstrated robust bacteriostasis against *E. coli* (86.17%) and *S. aureus* (93.65%) at pH 5.0 (Figure 6c), yielding strong PI fluorescence reminiscent of bacterial apoptosis (Figure 6b).

SEM revealed that the control groups of *E. coli* and *S. aureus* had burgeoning morphologies, indicating the vigorous vitality of both bacterial species (Figure 6d,e). CS‐Cin induced cell shrinkages of *E. coli* at pH 7.0 (Figure 6d), whereas *S. aureus* was unaffected (Figure 6e). Furthermore, both *E. coli* and *S. aureus* demonstrated extensive apoptotic morphologies, featuring rough, broken, and injured morphologies after treatment with CS‐Cin at pH 5.0 (Figure 6d,e). These results revealed that the antibacterial properties of CS‐Cin were highly dependent on pH, which may translate into respiration‐responsive bacteriostasis during the preservation of VFs.

### 2.6 pH‐Responsive Antioxidative Properties of CS‐Cin

**Figure 7:**
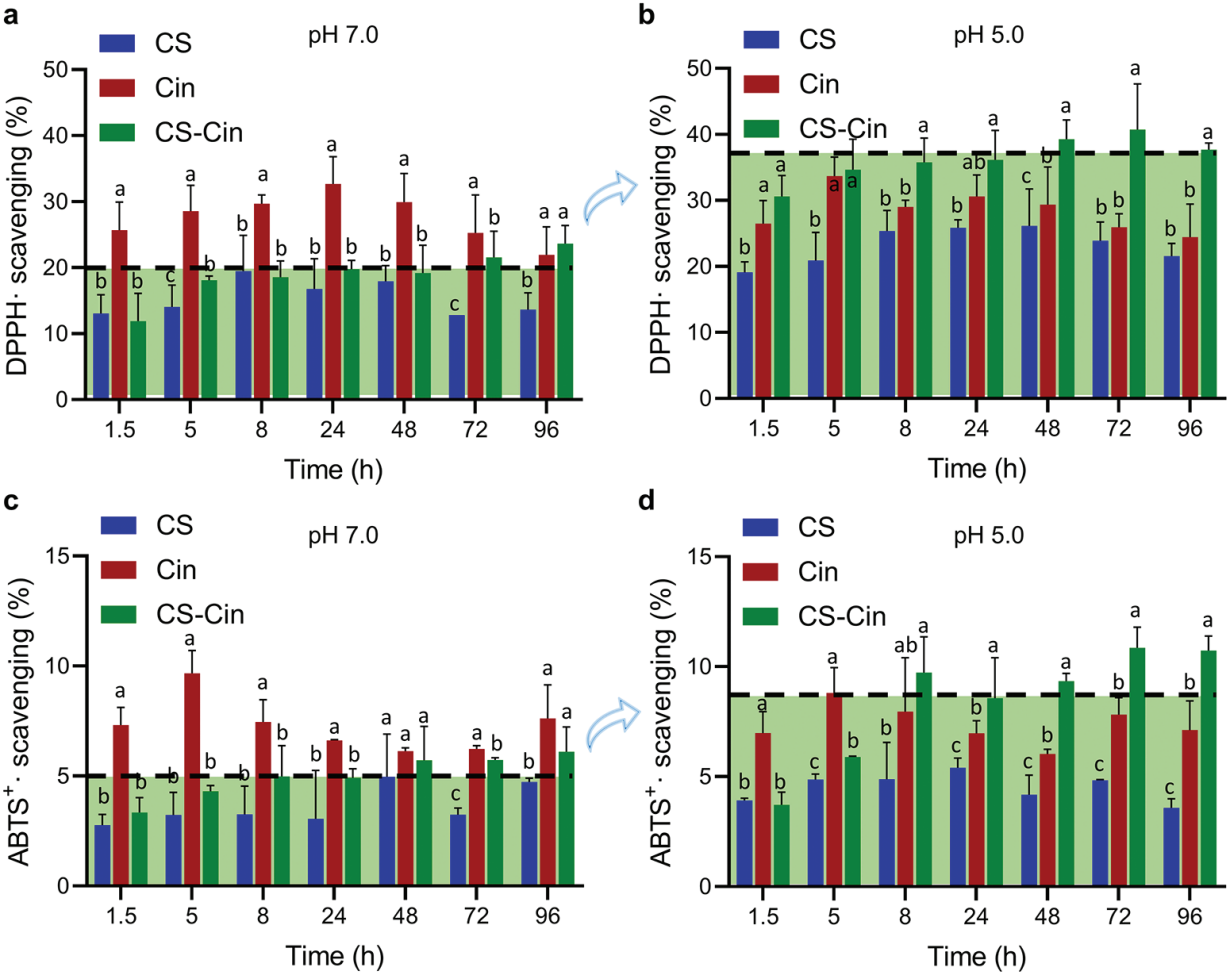
Antioxidative properties of CS‐Cin. a,b) DPPH• scavenging abilities at a) pH 7.0 and b) pH 5.0. c,d) ABTS^+^• scavenging abilities at c) pH 7.0 and d) pH 5.0. Different letters among counterparts indicate significant differences (*p* < 0.05).

The antioxidant properties of the preservatives were evaluated by the abilities of scavenging free radicals of DPPH• and ABTS^+•^, which were performed by immersing the preservatives in PBS, and their free radical‐scavenging abilities were tested with time. It can be seen that all of CS, Cin, and CS‐Cin at pH 7.0 had persistent antioxidative abilities throughout the 96‐h duration (**Figure** **7**a,c), while Cin had stronger DPPH• and ABTS^+^• scavenging abilities than CS and CS‐Cin due to the formation of stable phenoxy radicals via aromatic rings with hydroxyl groups.^[^ ^21^, ^24^ ^]^ By adjusting the incubation solution to pH 5.0, the free radical scavenging ability of CS improved to some extent (Figure 7b,d), which may be related to the transformation of ‐NH_2_ to ‐NH_3_^+^.^[^ ^25^ ^]^ In the meantime, the free radical‐scavenging abilities of Cin were more or less unaffected by the pH change owing to the relatively stable chemical structures under such weak variations in basicity (Figure 7b,d). However, the DPPH• and ABTS^+•^ scavenging abilities of CS‐Cin at pH 5.0, were improved by 90% and 60%, respectively, compared to those at pH 7.0 (Figure 6b,d). These results further indicated that CS‐Cin had pH‐responsive antioxidative abilities, which may promote respiration‐responsive prevention of peroxidation damage to VFs during long‐term storage.

## 3 Conclusion

Our study reveals a respiration‐triggered self‐saving strategy for the preservation of perishable VFs. With relevance to Schiff base reactions, respiration‐generated acid moisture triggered the release of antibacterial and antioxidant volatile Cin into the storage space. As a result, the respiration rate, peroxidation of the cytomembrane, and microbial growth of fresh produce were retarded, thereby leading to a 300% extension of the lifetime (from 2 days to 8 days from purchase) for broccoli and strawberries, which are characterized by high respiration intensities and fast rotting rates. Furthermore, both CS and Cin are biomolecules without food safety concerns, and this method is easy to implement, whereas CS is recyclable for repeated preservation with enhanced cost‐effectiveness. In addition, using volatile Cin as the preservative effectively circumvents the contact between preservatives and fresh food, which properly addresses the conformity concern in the case of the coating‐preservation method. The respiration‐responsive release of Cin provides a self‐saving approach toward the development of next‐generation smart preservation strategies for perishable food.

## 4 Experimental Section

### Materials

CS (with deacetylation degrees from 80−90% and molecule weights from 0.5–1.5 × 10^5^) was purchased from Golden‐Shell Pharmaceutical Co., Ltd. (Hangzhou, China). Cin with a purity ≥95% was purchased from Shanghai Macklin Biochemical Co., Ltd. (Shanghai, China). Ethanol (99.7% purity), sodium acetate (NaAc), and acetic acid (HAc) were purchased from Sinopharm Chemical Reagent Co., Ltd. (Shanghai, China). *Esherichia coli* (*E. coli)* (ATCC 8099) and I (*S. aureus)* (ATCC6538) were purchased from the American Type Culture Collection. All other reagents were analytical grade and were used as received unless otherwise noted.

### Synthesis of CS‐Cin

CS‐Cin was synthesized according to a previously reported method with minor modifications.^[^ ^26^ ^]^ Briefly, 3.0 g of CS was suspended in ethanol (50 mL) at room temperature (≈25 °C) under mild stirring (600 rpm) for 12 h. The Cin solutions (1 g Cin dissolved in 30 mL ethanol) were drop‐wise added to the CS dispersion at 45 °C, which were then stirred at 600 rpm for 8 h to obtain CS‐Cin. The CS‐Cin was washed several times with ethanol, and the unreacted Cin was removed by ethanol extraction for 12 h using a Soxhlet apparatus. Powdered CS‐Cin was obtained after drying in a vacuum at 50 °C for 24 h, which had a Cin loading of 246 mg g^−1^ CS and a Cin yield of 73.8%.

### Characterizations

Elemental analysis was determined by an elemental analyzer (Elementar vario MACRO cube) using the vacuum‐dried CS‐Cin powders, where the degree of substitution (DS) representing the percentage of amino sites grafted with Cin was calculated using the following equation^[^ ^27^ ^]^

(1) $$\mathrm{DS}=\frac{M_{N}R-\left(8-2\mathrm{DD}\right)M_{C}}{N_{C}M_{C}}$$

where DD is the deacetylation degree of Chitosan; M_N_ and M_C_ are the molecular weight of C and N, respectively; N_C_ is the number of carbon atoms in Cin; R is the molar ratio of C and N of substitutional derivatives. The cumulative released Cin at time *t* during preservation was calculated by the difference in DS of CS‐Cin at time *t* and that at time 0.

The morphologies of samples were examined by a VHX‐1000 digital microscope (Keyence Int. Trading Co. Ltd., Japan) and a high‐resolution scanning electron microscope (SEM, SU8100, Hitachi, Japan) after spray‐coated with gold for 120 s. To confirm the imidization reaction, the vacuum‐dried CS‐Cin powders were examined by LSCM (TCS SP2, Leica, Germany) excited at 488 nm, and the fluorescent images were obtained at 570–600 nm (red). The CS‐Cin powders were pressed with KBr into a pellet, and FT‐IR spectra were collected in the wavenumber range of 4000–500 cm^−1^ using an IS10 FT‐IR spectrophotometer (Nicolet, USA). XPS was studied using an XPS spectrometer (Thermo Scientific, USA) equipped with an X‐ray source bearing Al K Alpha radiation at 1486.6 eV. High‐resolution spectra of carbon (C1s) and nitrogen (N1s) were recorded at a pass energy of 60 eV. Spectral analysis was performed by the Thermo Advantage software.

### Preservative Effects of CS‐Cin

Strawberries and broccoli were used as the VF models for preservation by CS‐Cin during storage. The VFs were placed in an airtight, transparent PVC plastic container at 25 °C and 50% room humidity (RH), which were divided into four groups (4 replicates, each 100 g): a control group without preservatives and three tested groups with CS, Cin, and CS‐Cin as the sides without contact to the food, which were fixed at the center bottom of the containers. The colony count, malonaldehyde (MDA) content, rotting rate, respiration rate, and weight loss were measured to investigate the freshness of the VFs.

### Colony Count

The colony count was measured by the colony counting method during storage for 8 days. A total of 10 g of samples was diluted (1:10) with saline solution in a sterile stomacher bag, which was then homogenized for 5 min. After dilution, the samples were added to a plate count agar (PCA). The plates were incubated at 37 °C for 24 h and the colony‐forming units (CFU) were counted.

### MDA Content

The VFs were mixed with 5 mL trichloroacetic acid and centrifuged at 10 000 *g* and 4 °C for 20 min. Then, 2.0 mL thiobarbituric acid was added to the supernatants and the mixtures were boiled for 20 min. The MDA content was determined by measuring the absorbance at 450, 532, and 600 nm, which was expressed as µmol g^−1^ VFs:

(2) $$\mathrm{MDA}=\frac{(6.45\times \left(OD_{532}-OD_{600}\right)0.56\times OD_{450}\times V}{V_{s}\times m\times 1000}$$

where *m* represents the weight of the samples, *C* is the concentration of MDA (µmol L^−1^), *V* is the volume of the sample solution (mL), and *V_s_* is the volume of the solution used for determinations (mL).

### Rotting Rate

The rotting rate was characterized using a five‐point empirical scale:

(3) $$\mathrm{Rotting}\;\mathrm{rate}=\sum (\frac{d\times f}{N\times D})\times 100\%$$

where *f* is rot occurrence frequency, *N* is the total number of examined fruit (both healthy and infected), and *D* is the highest category of decay intensity observed on the empirical scale.

**Table:** 

Rotting rate	Area of infected fruit surface [%]
1	1–20
2	21–40
3	41–60
4	61–80
5	≥81 + concomitant sporulation

### Respiration Rate

The respiration rate was determined by the release of CO_2_. A 100 g of VFs were sealed in a 1 L glass pot for 1 h at room temperature, and the real‐time release of CO_2_ (mL kg^−1^ h^−1^) was determined by a gas analyzer (F‐940 Gas Analyzer, Felix, America).

### Weight Loss

Samples (five VFs per replication) were periodically weighed throughout the tested durations. The weight loss was expressed as the percentage of reduced weight at time *t* against the VF weight at time 0.

### pH‐Responsive Release of Cin

A sealed container with water spread over the bottom was filled with gas of different concentrations of CO_2_ (5%, 10%, 15%, and 20%). The pH of the liquid in the container was determined by a pH test paper. The release rate of Cin was determined at 292 nm by a UV–vis spectrometer.

### Antibacterial Properties of CS‐Cin

*E. coli* and *S. aureus* representative of Gram‐negative and Gram‐positive bacteria, respectively, were used to evaluate the antibacterial properties of CS‐Cin. The bacteria were inoculated in Luria–Bertani (LB) broth medium and cultured overnight at 37 °C with unremitted shaking at 150 rpm. Then, 100 µL of bacteria suspensions were transferred to 10 mL of LB medium and incubated until reaching the logarithmic phase. Subsequently, the inoculums were diluted to 10^5^ CFU ml^−1^ with PBS to obtain a bacterial suspension. 100 µL of the bacterial suspension were plated on precooled agar medium treated with sodium acetate buffer solution (pH 5.0 or 7.0) containing CS‐Cin (1.25 mg mL^−1^), CS (1 mg mL^−1^), or Cin (0.25 mg mL^−1^) for 24 h. The bacteria without any treatment were used as a control. The inhibition of bacteria growth by CS‐Cin was determined by the colony counting method, which was expressed as:

(4) $$\begin{array}{c} \mathrm{Inhibitory}\;\mathrm{rate}\;=\;\frac{\mathrm{CFU}\;\mathrm{of}\;\mathrm{control}--\mathrm{CFU}\;\mathrm{of}\;\mathrm{experimental}\;\mathrm{group}}{\mathrm{CFU}\;\mathrm{of}\;\mathrm{control}}\times 100\% \end{array}$$

### Observation of Bacteria by SEM

The morphologies of bacteria were observed by SEM. Briefly, the bacteria were allowed to grow in LB medium to reach the mid‐log phase and then cultured with CS‐Cin at 37 °C for 24 h. After fixated with glutaraldehyde (2.5%) overnight at 4 °C, the microbes were carefully washed with PBS thrice and dehydrated with a solution of graded ethanol concentrations (30%, 50%, 70%, 80%, 90%, 100%, each 10 min). After free‐drying, the samples were sputter‐coated with gold and imaged using the same SEM as above.

### DPPH• Scavenging Activity

The dispersions of CS, Cin, and CS‐Cin were prepared by suspending samples into sodium acetate buffer solutions (pH 5.0 or 7.0). Then, 50 µL of sample solution was subjected to react with 150 µL of 0.35 mM DPPH• solution (in 95% ethanol), which was then kept in the darkness for 30 min. Subsequently, the absorbance of the mixture was measured with a MULTISKAN microplate reader (ThermoFisher Scientific Co., Madison, USA) at 517 nm. The DPPH• scavenging rate was calculated as:

(5) $$\mathrm{DPPH}•\;\mathrm{scavenging}\;\mathrm{rate}\;=\;\frac{A_{b}-\left(A_{s}-A_{c}\right)}{A_{b}}\times 100\%$$

where *A_b_* is the absorbance of the sodium acetate buffer solution added with DPPH• working solution, *A_c_* is the absorbance of the sample solution, and *A_s_* is the absorbance of the sample solution added with DPPH• working solution.

### ABTS•^+^ Scavenging Activity

Equal volumes of the solutions of ABTS•^+^ (7.4 mm) and potassium persulfate (2.6 mm) were mixed and allowed for reactions for 12 h at 25°C in the darkness to obtain a stock solution. Then, 50 µL of sample solution was brought to react with 150 µL ABTS•^+^ and kept in the darkness for 6 min. The absorbance at 414 nm was measured with the same microplate reader as above. The ABTS•^+^ scavenging rate was calculated as:

(6) $${\mathrm{ABTS}•}^{+}\mathrm{scavenging}\;\left(\%\right)=100\;\times \;\frac{A_{b}-\left(A_{s}-A_{c}\right)}{A_{b}}$$

where *A_b_* is the absorbance of the sodium acetate buffer solution added with ABTS•^+^ working solution, *A_c_* is the absorbance of the sample solution, and *A_s_* is the absorbance of the sample solution added with ABTS•^+^ working solution.

### Statistical Analysis

All experiments were conducted in at least three independent replicates following completely randomized designs, and results were expressed as the mean ± standard deviation (n = 3). One‐way analysis of variance (ANOVA) was performed by Duncan's test using SPSS 26.0 software package (IBM, New York).

## Conflict of Interest

The authors declare no conflict of interest.

## Supporting information

Supporting Information

## Data Availability

The data that support the findings of this study are available from the corresponding author upon reasonable request.
