# Supplementary material for: Respiration‐Triggered Release of Cinnamaldehyde from a Biomolecular Schiff Base Composite for Preservation of Perishable Food
Source: Adv Sci (Weinh). 2023 Dec 21;11(9):2306056. doi: 10.1002/advs.202306056 (PMC10916653; doi:10.1002/advs.202306056)
Supplement: Supplementary file 1 — Supporting Information [file ADVS-11-2306056-s001.pdf]

ADVANCED SCIENCE
Open Access

Supporting Information

for *Adv. Sci.*, DOI 10.1002/advs.202306056

Respiration-Triggered Release of Cinnamaldehyde from a Biomolecular Schiff Base Composite for Preservation of Perishable Food

*Fei Liu, Lingyun Kuai, Chen Lin, Maoshen Chen, Xing Chen, Fang Zhong and Tao Wang**

Supporting Information for:

Respiration-triggered Release of Cinnamaldehyde from a Biomolecular Schiff Base Composite for Preservation of Perishable Food

*Fei Liu, Lingyun Kuai, Chen Lin, Maoshen Chen, Xing Chen, Fang Zhong, and Tao Wang**

F. L., L. K., C. L., M. C., X. C., F. Z., T. W.
School of Food Science and Technology, Jiangnan University, Wuxi 214122, China
C. L., T. W.
National Engineering Research Center for Cereal Fermentation and Food Biomanufacturing, Jiangnan University, Wuxi 214122, China
F. L., L. K., M. C., F. Z.
Science Center for Future Foods, Jiangnan University, Wuxi 214122, China
International Joint Laboratory on Food Safety, Jiangnan University, Wuxi 214122, China
F. L., F. Z.
Jiaxing Institute of Future Food, Jiaxing 314050, China

*Corresponding author: twang3813@gmail.com (T. W.).

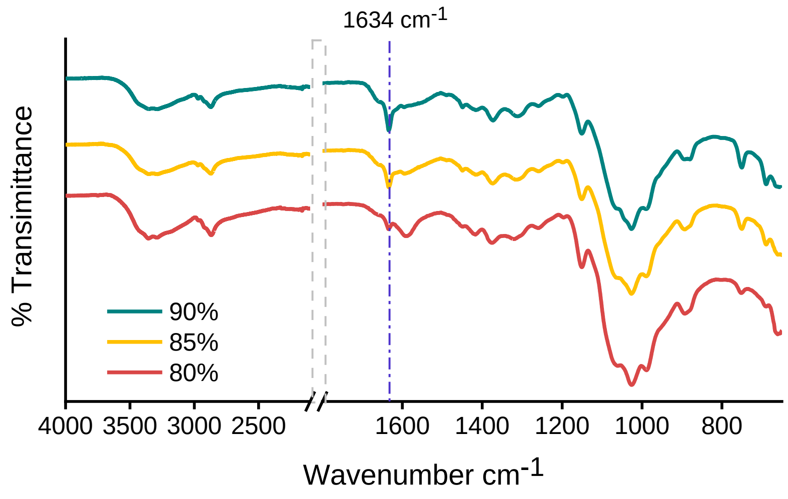

Figure S1. FT-IR spectra of CS-Cin prepared from CS of different levels of

deamidization (80%–90%).

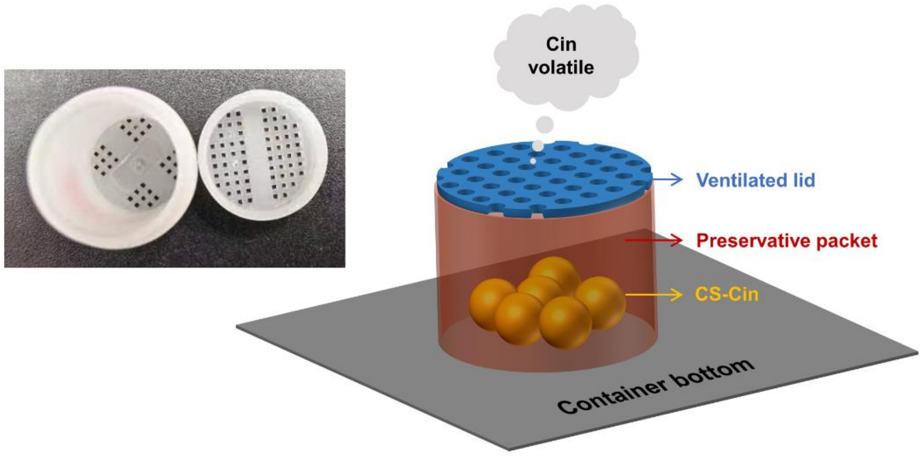

Figure S2. Photograph of the ventilated preservative packet and schematics of its layout in the preservation container.

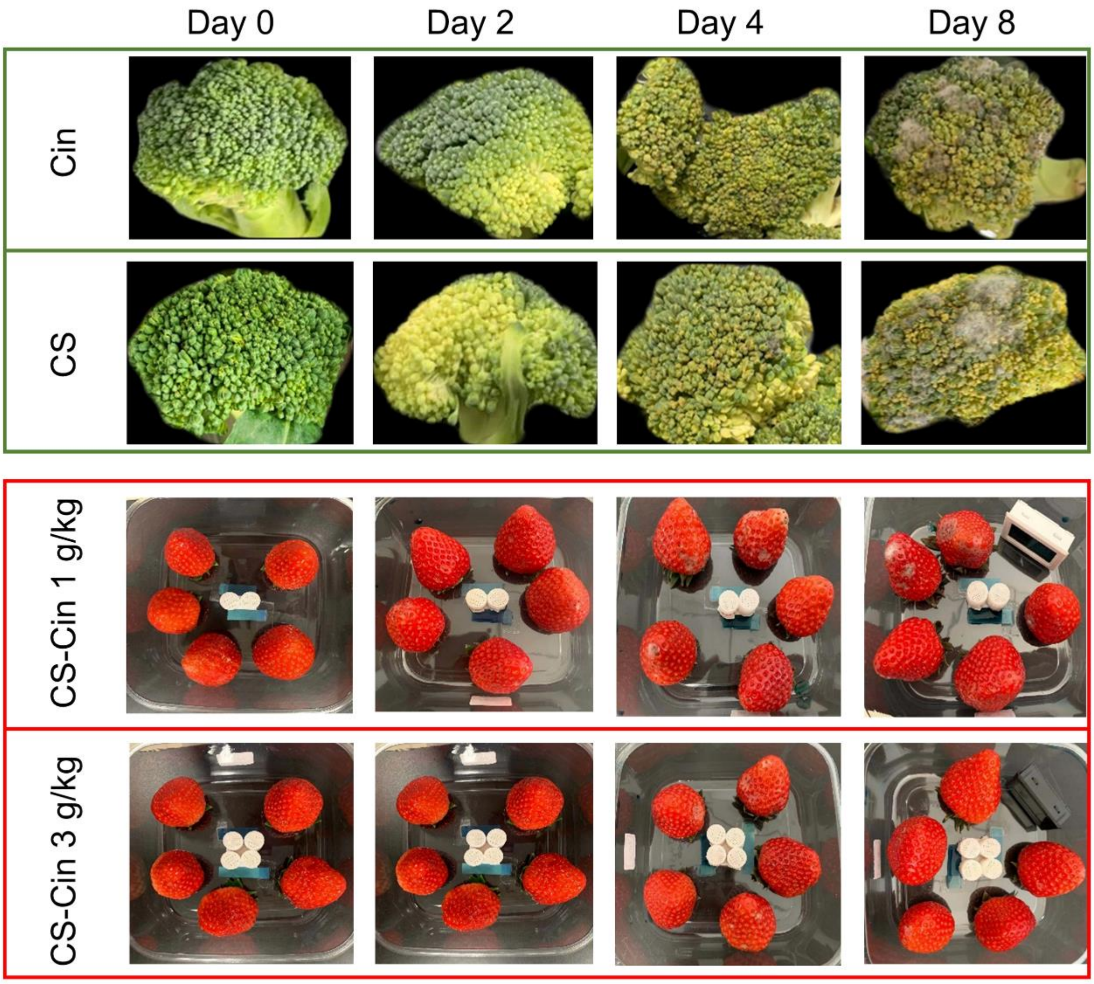

Figure S3. Optical images of VFs stored for different durations.

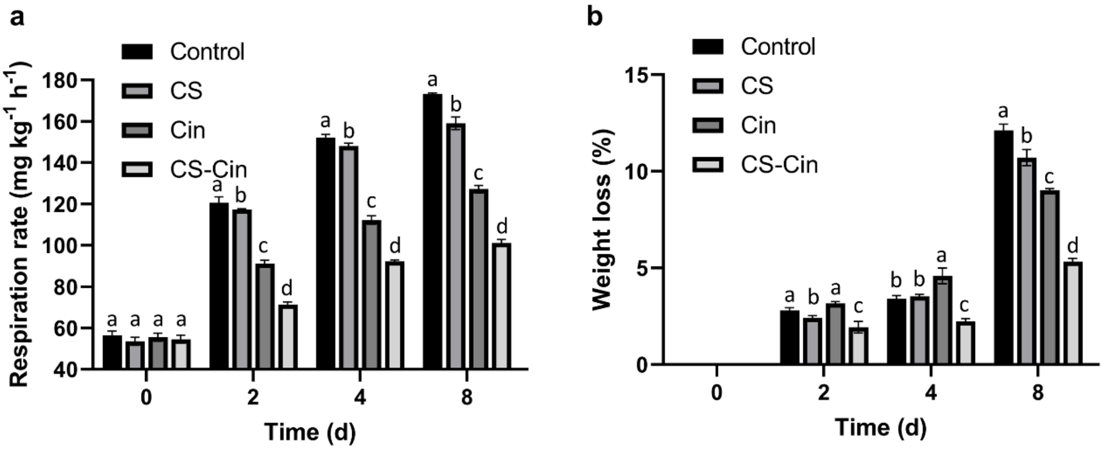

Figure S4. Respiration rate and weight loss of broccoli among the groups of control, CS, Cin, and CS-Cin (2 g/kg). Different letters among counterparts indicate significant differences ($p < 0.05$).

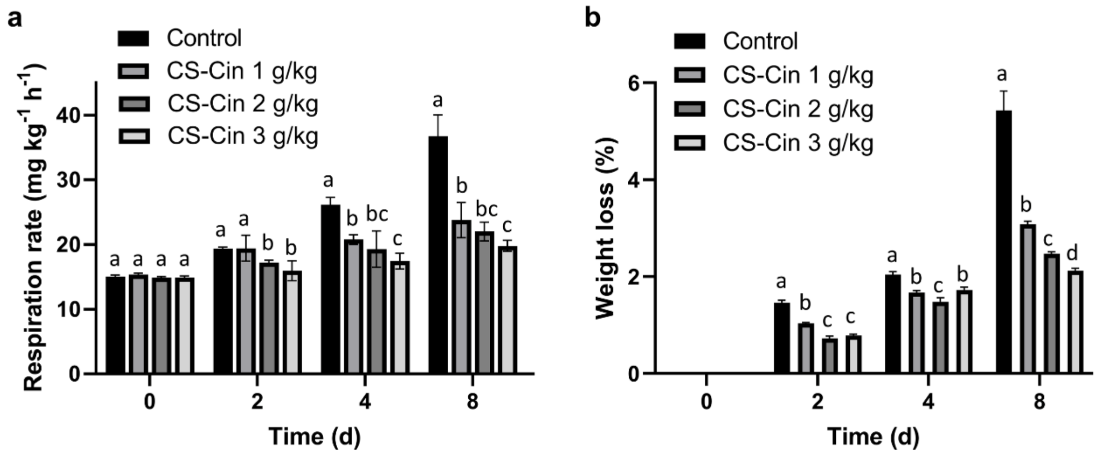

Figure S5. Respiration rate and weight loss of strawberry preserved by CS-Cin of different dosages. Different letters among counterparts indicate significant differences ($p < 0.05$).

Table S1. Percentage of peak areas of C1s in CS and CS-Cin calculated by peak fitting of high-resolution XPS spectra.

	C1s peak area%		
	C-C, C-H	C-O-C, C-N	O=C-NH
CS	68.44	20.50	11.06
CS-Cin	54.78	34.66	10.56
